# Effects of melatonin alone or in combination with Bio-Oss® on early osteoblastic activity: An *in vitro* study

**DOI:** 10.1016/j.jobcr.2026.101561

**Published:** 2026-10-10

**Authors:** Christiane Colombo dos Santos, Daiane Cristina Peruzzo, Juliana Ramacciato, Ana Cláudia Garcia Rosa

**Affiliations:** a Afya Faculty of Medical Sciences, School of Dentistry, Palmas, Tocantins, Brazil; b São Leopoldo Mandic School of Dentistry, Postgraduate Program, Campinas, São Paulo, Brazil; c São Leopoldo Mandic School of Dentistry, Department of Pharmacology, Campinas, São Paulo, Brazil; d Federal University of Tocantins, School of Medicine, Department of General Pathology, Palmas, Tocantins, Brazil

**Keywords:** Melatonin, Osteoblast-like cells, Bone substitutes, Cell proliferation, Type I collagen

## Abstract

**Background:**

Melatonin modulates bone metabolism and enhances osteogenic activity; however, whether these effects are maintained when combined with xenogeneic bone substitutes during early osteoblastic responses remains unclear. This study investigated the effects of melatonin alone and combined with the xenogeneic bone substitute Bio-Oss® on early osteoblastic activity *in vitro*.

**Methods:**

Human osteoblast-like cells (SaOS-2) were allocated to four groups: control, melatonin (0.1 mM), Bio-Oss® (10 mg/mL), and melatonin plus Bio-Oss®. Three independent biological experiments were performed. Cell proliferation (Trypan blue exclusion), viability (MTT assay), and type I collagen secretion (ELISA) were assessed at 24 h, 72 h, and 7 days. Data were analyzed using the Kruskal-Wallis test followed by Dunn's post hoc test (*p* < 0.05).

**Results:**

Proliferation and viability increased over time in all groups. At 7 days, Bio-Oss®-containing groups showed significantly reduced proliferation compared with the control group (*p* < 0.05), whereas the melatonin-only group did not differ significantly (*p* > 0.05). Cell viability was lower in Bio-Oss®-containing groups at 72 h and in the melatonin plus Bio-Oss® group at 7 days (*p* < 0.05). Type I collagen secretion did not differ significantly among groups at any time point (*p* > 0.05). Melatonin alone did not differ significantly from the control, whereas its combination with Bio-Oss® did not enhance early osteoblastic activity.

**Conclusion:**

Combining melatonin with Bio-Oss® did not enhance early osteoblastic activity in this *in vitro* model. These findings provide useful information for future studies investigating combinations of bioactive molecules and xenogeneic bone substitutes in bone regeneration.

## 1 Introduction

Bone regeneration is a complex process that relies on coordinated interactions among cells, the extracellular matrix, and biochemical mediators. In dental practice, biomaterials are commonly utilized to support bone formation and improve regenerative outcomes. Xenogeneic bone substitutes, such as Bio-Oss® (deproteinized bovine bone), are frequently selected for their biocompatibility and osteoconductive characteristics. These substitutes serve as three-dimensional scaffolds that preserve the mineral architecture of bone and support osteogenic cell adhesion, migration, and activity.^1^ Among the available bone graft materials, Bio-Oss® is one of the most extensively investigated xenogeneic bone substitutes in oral and maxillofacial regeneration. Its widespread clinical use and well-documented osteoconductive properties make it an appropriate experimental model for evaluating biomolecule–biomaterial interactions.^1^

Bioactive molecules have been investigated for their ability to modulate cellular responses during bone regeneration. Melatonin (N-acetyl-5-methoxytryptamine), primarily known for its function in circadian rhythm regulation, has recently been identified as a regulator of bone metabolism 2, 3, 4. Experimental studies have demonstrated that melatonin promotes osteoblastic differentiation, reduces oxidative stress, and modulates the OPG/RANKL axis, a key mediator of osteoclastogenesis and bone remodeling 2, 3, 4. *In vitro* studies have also demonstrated that melatonin increases the expression of osteogenic markers, including type I collagen (COL-I) and osteopontin (OPN), while enhancing extracellular matrix mineralization.^5^ These findings suggest that melatonin may enhance osteogenic activity and support its further investigation in regenerative strategies. Additionally, recent preclinical studies have reported favorable effects on bone regeneration and osseointegration when melatonin is associated with xenogeneic bone graft materials 6, 7, 8, 9.

Despite growing evidence supporting the osteogenic effects of melatonin, it remains unclear whether these beneficial effects are preserved in the presence of xenogeneic bone substitutes 2, 3, 4, 5, 6, 7, 8, 9. Most previous studies have evaluated melatonin alone or have focused on pre-osteoblastic models, *in vivo* bone regeneration, or osteogenic differentiation rather than on biomaterial-melatonin interactions during the early phase of osteoblastic activity 5, 6, 7, 8, 9. Consequently, the influence of combining melatonin with xenogeneic bone substitutes on early osteoblastic responses remains poorly understood. This represents an important knowledge gap because early cellular responses are critical determinants of subsequent bone regeneration.

Therefore, the present study was designed to investigate whether the osteogenic effects of melatonin are maintained when combined with Bio-Oss®, one of the most widely used xenogeneic bone substitutes in oral regeneration. Specifically, we evaluated early osteoblastic activity by assessing cell proliferation, viability, and type I collagen secretion in an *in vitro* SaOS-2 model.

## 2 Methods

### 2.1 Ethics statement

This study was conducted using an immortalized cell line, with no involvement of human participants or animals. Accordingly, the Research Ethics Committee of Faculdade São Leopoldo Mandic granted exemption from ethical review (protocol no. 2019/0184).

### 2.2 Cell culture

The human osteoblast-like cell line SaOS-2 (ATCC HTB-85; American Type Culture Collection, Manassas, VA, USA) was utilized. Cells were cultured in α-MEM (alpha-modified Minimum Essential Medium) supplemented with 10% fetal bovine serum (FBS; Cultilab®, Campinas, SP, Brazil) and 1% antibiotic-antimycotic solution (Sigma-Aldrich, St. Louis, MO, USA).

Cell cultures were maintained at 37 °C in a humidified atmosphere containing 5% CO_2_ and 95% air. The culture medium was replaced every 2-3 days. Cell growth was monitored using phase-contrast microscopy.

Cells between passages 4 and 8 were selected to ensure phenotypic stability. Cultures were maintained until reaching 80-90% confluence before experimental seeding. Cultures exhibiting morphological changes indicative of senescence or contamination were discarded.

### 2.3 Melatonin preparation

Synthetic melatonin (N-acetyl-5-methoxytryptamine; Huanggang Saikang Pharmaceutical Co., Ltd.) was dissolved in absolute dimethyl sulfoxide (DMSO) to prepare a 100 mM stock solution. The solution was sterilized by filtration through a 0.22 μm membrane.

For experimental use, the stock solution was diluted in α-MEM to achieve a final melatonin concentration of 0.1 mM and a final DMSO concentration of 0.1% (v/v). The control group was exposed to the same DMSO concentration to control for vehicle effects.

Working solutions were freshly prepared before each experiment and protected from light.

### 2.4 Xenogeneic bone substitute

The xenogeneic bone substitute Bio-Oss® (particle size 250-1000 μm; Geistlich Biomaterials, Wolhusen, Switzerland) was handled under aseptic conditions and used as supplied by the manufacturer. The material was weighed with an analytical balance and added directly to culture wells during cell seeding, without prior hydration.

Granules were evenly distributed at the bottom of each well to achieve a final concentration of 10 mg/mL, facilitating direct contact between cells and biomaterial.

### 2.5 Experimental design

Cells were cultured in 24-well polystyrene plates (growth area approximately 1.9 cm^2^ per well) with a final medium volume of 1 mL per well. For all cellular assays, cells were seeded at a density of 1.1 × 10^4^ cells/cm^2^. The experimental groups were organized as detailed in Table 1.

**Table 1:** Composition of the experimental groups.

Group	Basal medium (α-MEM)	Melatonin (0.1 mM)	Bio-Oss® (10 mg/mL)	Osteogenic factors*
Control (C)	Yes	No	No	Yes
MLT	Yes	Yes	No	Yes
BO	Yes	No	Yes	Yes
MLT + BO	Yes	Yes	Yes	Yes

**Legend:** *β-glycerophosphate (10 mM) and ascorbic acid (50 μg/mL).

All experiments were performed in three independent biological experiments conducted on separate occasions using independently cultured cells. Within each biological experiment, all experimental conditions were evaluated in triplicate wells (technical replicates).

### 2.6 Cell proliferation assay

Cell proliferation was evaluated using the Trypan blue exclusion method at 24 h, 72 h, and 7 days. Cell counts were performed for each technical replicate, and the mean value of the triplicate wells was used for statistical analysis.

Cells were seeded at a density of 1.1 × 10^4^ cells/cm^2^. At each time point, cells were enzymatically detached using trypsin-EDTA, centrifuged, and resuspended in 1 mL of medium. An aliquot was combined with Trypan blue (1:1) and analyzed in a Neubauer chamber using phase-contrast microscopy. Only viable (unstained) cells were enumerated.

### 2.7 Cell viability assay (MTT)

Cell viability was determined using the MTT assay. Cells were seeded at a density of 1.1 × 10^4^ cells/cm^2^ and, following the experimental period, incubated with MTT solution (5 mg/mL) for 3 h at 37 °C.

After incubation, 100 μL of DMSO was added to solubilize the formazan crystals. The resulting solution was then transferred to a new 96-well plate, and absorbance was measured at 590 nm using a microplate reader to avoid interference from Bio-Oss® granules.

To minimize optical interference from the biomaterial, background controls containing only Bio-Oss® and culture medium (without cells) were included and processed under identical conditions as the experimental samples. The resulting values were subtracted from those of the corresponding experimental groups.

Absorbance measurements were obtained from each technical replicate, and the mean value was used for statistical analysis.

### 2.8 Total protein quantification

Total protein content was quantified at 24 h, 72 h, and 7 days using the Lowry method.^10^ Following PBS washing, cells were lysed by repeated freeze-thaw cycles, and protein concentration was determined by spectrophotometry at 680 nm using a bovine serum albumin (BSA) standard curve.

### 2.9 ELISA for type I collagen

Type I collagen secretion was quantified using an ELISA kit (R&D Systems, USA) in accordance with the manufacturer's instructions. Culture supernatants were collected and centrifuged at 5000 × g for 15 min at 4 °C.

Absorbance was measured at 450 nm. Results were expressed as pg/mL, normalized to the total protein content of each well, and averaged across the triplicate wells for each biological experiment.

### 2.10 Data analysis

Cell proliferation, cell viability, total protein content, and type I collagen secretion were evaluated at 24 h, 72 h, and 7 days.

Data normality was initially assessed. Because a non-normal distribution was observed, differences among groups were analyzed using the Kruskal-Wallis test followed by Dunn's post hoc test for multiple comparisons.

Statistical analyses were performed using SPSS version 23 (SPSS Inc., Chicago, IL, USA) and BioEstat 5.0 (Mamirauá Foundation, Belém, PA, Brazil). Data from the three independent biological experiments were analyzed using the mean values obtained for each experimental condition. Statistical significance was established at *p* < 0.05.

## 3 Results

The results of the present study are summarized in Table 2.

**Table 2:** Cell proliferation, viability, and type I collagen secretion.

Group	Time	Proliferation (×10^4^ cells/mm^2^)	Viability (AU)	Collagen I (pg/mL)
Control	24 h	0.28 ± 0.08	0.151 ± 0.013	1034.62 ± 156.41
Control	72 h	0.39 ± 0.09	0.252 ± 0.023	1320.00 ± 259.52
Control	7 d	2.73 ± 0.42	0.597 ± 0.039	1518.40 ± 86.57
Bio-Oss®	24 h	0.19 ± 0.06	0.130 ± 0.014	1013.18 ± 51.57
Bio-Oss®	72 h	0.25 ± 0.03	0.199 ± 0.004	1056.83 ± 84.82
Bio-Oss®	7 d	1.73 ± 0.23	0.424 ± 0.062	1291.61 ± 280.11
Melatonin	24 h	0.20 ± 0.07	0.135 ± 0.009	1019.23 ± 194.50
Melatonin	72 h	0.28 ± 0.06	0.214 ± 0.008	1133.05 ± 233.35
Melatonin	7 d	2.20 ± 0.20	0.568 ± 0.038	1185.65 ± 189.28
MLT + BO	24 h	0.24 ± 0.02	0.120 ± 0.003	1024.58 ± 214.40
MLT + BO	72 h	0.32 ± 0.05	0.134 ± 0.006	939.54 ± 156.88
MLT + BO	7 d	1.47 ± 0.31	0.304 ± 0.060	1162.60 ± 231.66

**Legend: Legend:** Data are expressed as mean ± standard deviation (SD) from three independent biological experiments (n = 3). Statistical analyses were performed using the Kruskal-Wallis test followed by Dunn's post hoc test**.** Statistical significance was set at *p* < 0.05.

### 3.1 Cell proliferation

Cell proliferation was influenced by incubation time. In all experimental groups, proliferation values at 7 days were significantly higher than those at 24 h (*p* < 0.05). The 72-h time point showed intermediate values. No significant differences in cell proliferation were observed among experimental groups at 24 or 72 h (*p* > 0.05). At 7 days, groups treated with Bio-Oss®, either alone or combined with melatonin, demonstrated lower proliferation compared to the control group (*p* < 0.05). In contrast, the melatonin-only group exhibited proliferation values similar to the control (*p* > 0.05) (Fig. 1).

**Fig. 1:**
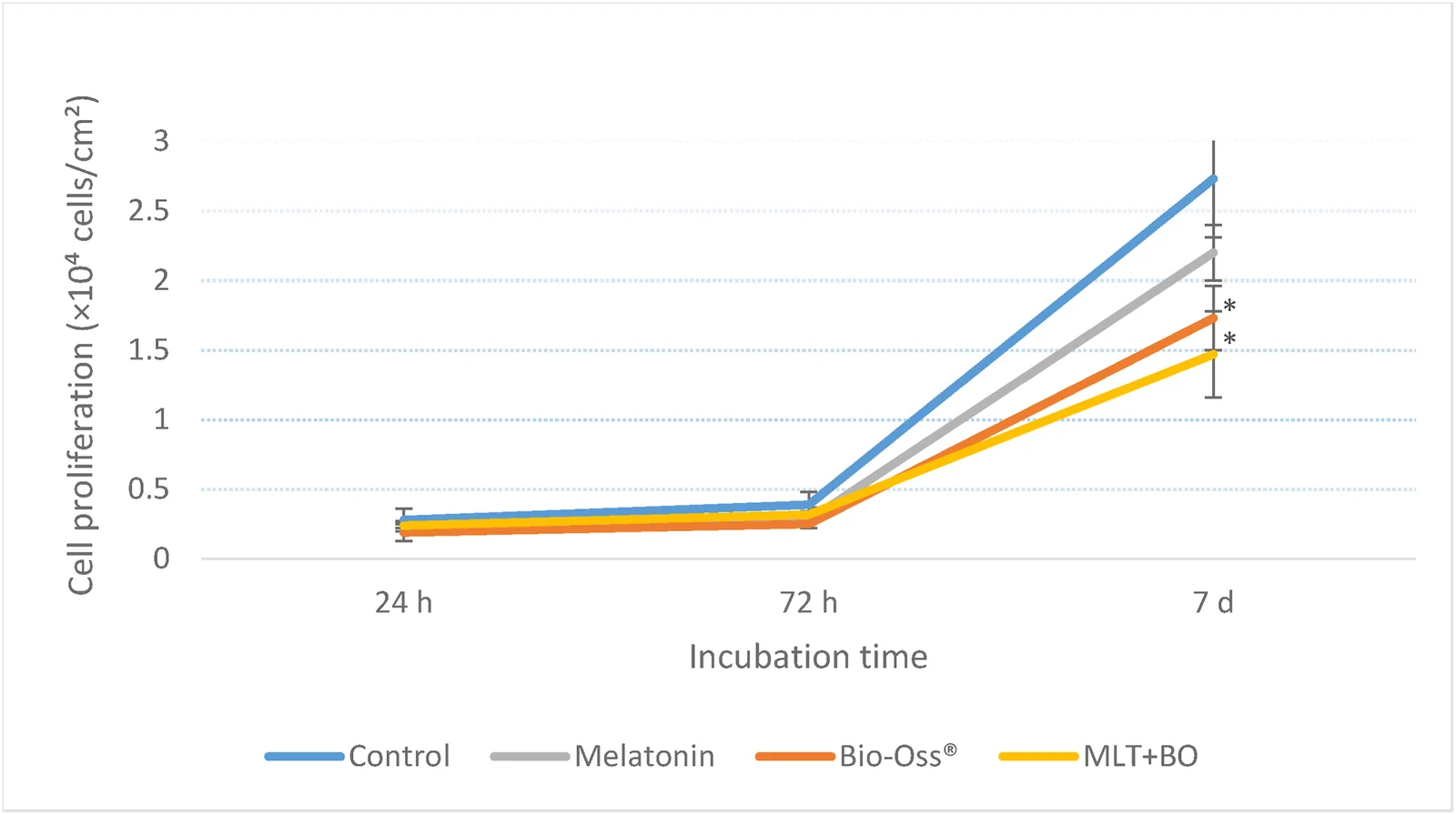
Cell proliferation **Legend:** Cell proliferation of SaOS-2 osteoblast-like cells after 24 h, 72 h, and 7 days of incubation in the control (C), melatonin (MLT), Bio-Oss® (BO), and melatonin + Bio-Oss® (MLT + BO) groups. Data are expressed as mean ± standard deviation (SD) from three independent biological experiments (n = 3). Statistical comparisons were performed using the Kruskal-Wallis test followed by Dunn's post hoc test**.** *Statistically significant difference compared with the control group at the same experimental time point (*p* < 0.05).

### 3.2 Cell viability

Cell viability increased with longer incubation, showing significantly higher values at 7 days compared to 24 h (*p* < 0.05). The 72-h time point showed intermediate viability values between those observed at 24 h and 7 days. No significant differences were observed between groups at 24 h (*p* > 0.05). At 72 h, the control group exhibited higher viability than both the Bio-Oss® and MLT + BO groups (*p* < 0.05), while the melatonin-only group did not differ from the control (*p* > 0.05).

After 7 days, the control, melatonin-only, and Bio-Oss® groups demonstrated similar viability (*p* > 0.05). In contrast, the MLT + BO group exhibited significantly lower viability compared to the control group (*p* < 0.05) (Fig. 2).

**Fig. 2:**
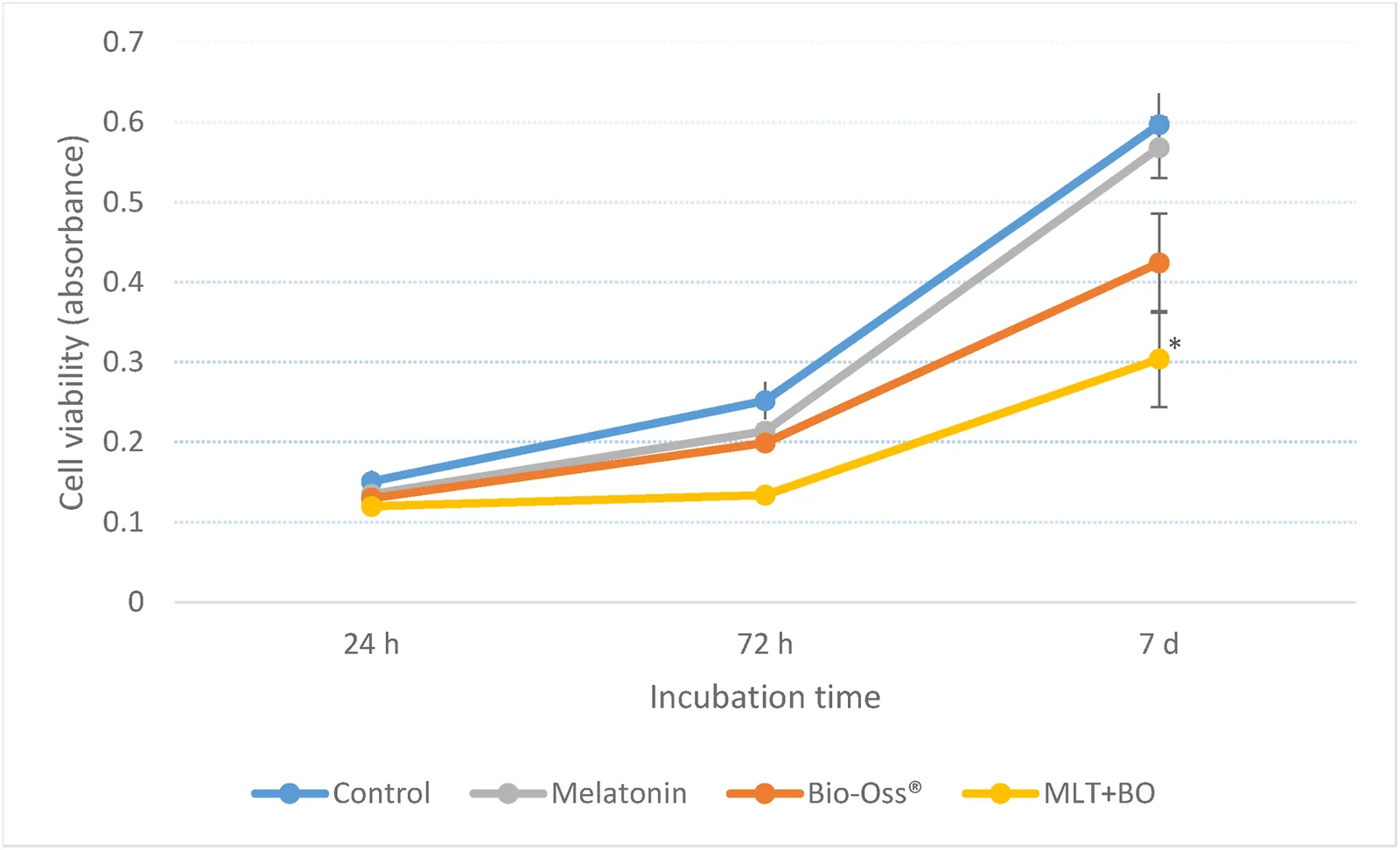
Cell Viability **Legend:** Cell viability of SaOS-2 osteoblast-like cells after 24 h, 72 h, and 7 days of incubation in the control (C), melatonin (MLT), Bio-Oss® (BO), and melatonin + Bio-Oss® (MLT + BO) groups. Data are expressed as mean ± standard deviation (SD) from three independent biological experiments (n = 3). Statistical comparisons were performed using the Kruskal-Wallis test followed by Dunn's post hoc test**.** *Statistically significant difference compared with the control group at the same experimental time point (*p* < 0.05).

### 3.3 Collagen type I secretion

No statistically significant differences in type I collagen secretion were detected among the experimental groups at any evaluated time point (24 h, 72 h, and 7 days) (*p* > 0.05). A trend toward increased type I collagen secretion over time was observed in some groups; however, these differences were not statistically significant (Fig. 3).

**Fig. 3:**
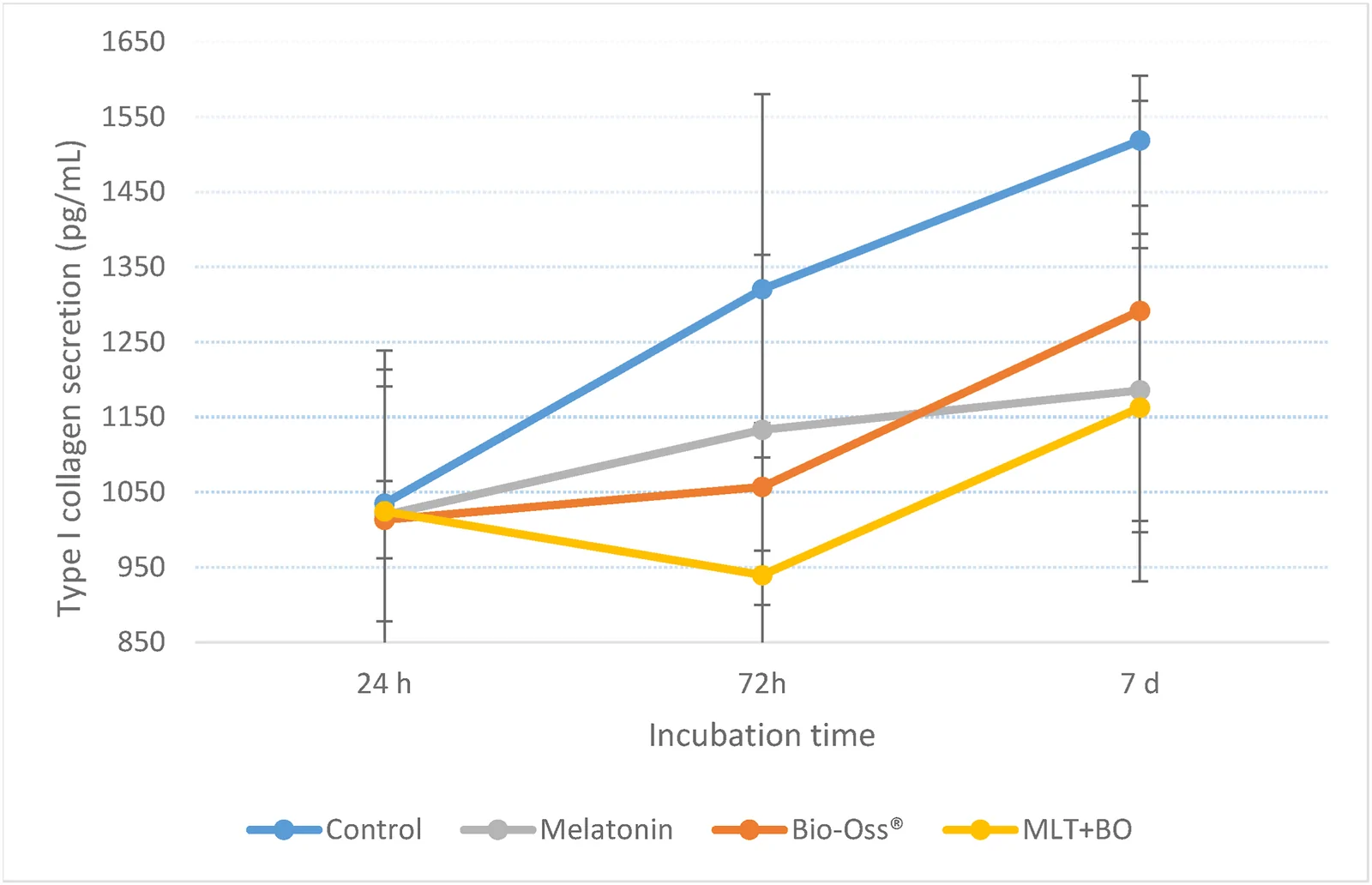
Type I Collagen Secretion **Legend:** Type I collagen secretion by SaOS-2 osteoblast-like cells after 24 h, 72 h, and 7 days of incubation in the control (C), melatonin (MLT), Bio-Oss® (BO), and melatonin + Bio-Oss® (MLT + BO) groups. Data are expressed as mean ± standard deviation (SD) from three independent biological experiments (n = 3). Statistical comparisons were performed using the Kruskal–Wallis test followed by Dunn's post hoc test. No statistically significant differences were observed among groups at any experimental time point (*p* > 0.05).

## 4 Discussion

The present study examined the effects of melatonin, both independently and in combination with the bone substitute Bio-Oss®, on early osteoblastic activity in human SaOS-2 cells *in vitro*. While melatonin has been widely reported to enhance osteogenic activity, particularly in pre-osteoblastic models,^5^^,^^6^^,^^8^ such stimulatory effects were not clearly observed under the current experimental conditions.

A melatonin concentration of 0.1 mM was selected based on previous studies demonstrating biological activity in *in vitro* models. Dalla-Costa et al. (2020) reported that concentrations ranging from 0.01 mM to 1 mM enhanced osteogenic marker expression and promoted mineralized matrix formation in murine pre-osteoblastic cells (MC3T3-E1), which are typically more responsive to osteogenic stimuli.^5^ These findings provided the rationale for the concentration used in this study.

This investigation focused on early events of osteoblastic activity, including cell proliferation, metabolic viability, and type I collagen secretion, which are essential for subsequent extracellular matrix formation and mineralization. Overall, the combination of melatonin with Bio-Oss® did not enhance the evaluated parameters under the present experimental conditions.

Regarding cell proliferation, Bio-Oss® alone or in combination with melatonin was associated with lower proliferation at 7 days compared with the control group. However, this finding warrants cautious interpretation. The reduced proliferation observed in Bio-Oss®-containing groups may reflect alterations in cell distribution, adhesion dynamics, or spatial constraints imposed by the particulate biomaterial, rather than a direct inhibitory effect on cell growth. In contrast, melatonin alone resulted in proliferation values comparable to the control, indicating no measurable effect on early cell expansion under these experimental conditions.

For cell viability, groups containing Bio-Oss®, particularly when combined with melatonin, exhibited lower values than the control at intermediate and late time points. This outcome may be related to the physical characteristics of the biomaterial, such as its particulate structure, which can influence cell distribution, available surface area for growth, and nutrient diffusion within the culture microenvironment. Melatonin alone demonstrated viability comparable to the control throughout the experimental period, indicating the absence of cytotoxic effects under these conditions. Another possible explanation is that Bio-Oss® may adsorb melatonin onto its surface, thereby reducing its local availability to the cells. However, this hypothesis was not directly investigated in the present study and therefore remains speculative.

Despite changes in proliferation and viability, type I collagen secretion did not differ significantly between groups or across time points. These findings suggest that early osteoblastic activity, as reflected in extracellular matrix synthesis, was maintained across experimental conditions despite alterations in proliferative and metabolic parameters. Such discrepancies may result from differences in experimental design, including the stage of cellular differentiation, the presence or absence of biomaterials, and the specific biological endpoints evaluated.

The present findings partially contrast with previous reports demonstrating beneficial effects of melatonin on osteogenic activity in both *in vitro* and *in vivo* models 5, 6, 7, 8. These discrepancies may reflect methodological differences, including the cell type employed, the presence or absence of biomaterials, the experimental conditions, and the biological endpoints evaluated.

Pre-osteoblastic cells generally exhibit greater responsiveness to osteogenic stimuli, whereas more differentiated osteoblastic cells, such as the SaOS-2 lineage, may show more limited responses. Therefore, the limited responsiveness observed in the present study may, at least in part, be related to the more differentiated phenotype of the SaOS-2 cell line. Additionally, *in vitro* models do not fully replicate the complexity of the *in vivo* environment, which involves cellular interactions, inflammatory responses, and angiogenesis.^6^

The presence of the biomaterial may also have contributed to the differences observed between experimental groups. Xenogeneic biomaterials possess high surface area and porosity, characteristics that may influence the adsorption of molecules from the culture medium as well as cell adhesion and distribution. However, these potential biomaterial–molecule interactions were not directly evaluated in the present study. Therefore, whether such mechanisms contributed to the observed biological responses remains to be determined.

The present study contributes to the current understanding of the combined effects of melatonin and xenogeneic bone substitutes by evaluating their influence on early osteoblastic responses using the human osteoblast-like cell line SaOS-2. The use of complementary outcome measures, including cell proliferation, viability, and type I collagen secretion, provided a broader assessment of early cellular behavior. Although no enhancement of early osteoblastic activity was observed under the experimental conditions evaluated, these findings provide useful information for future studies investigating combinations of bioactive molecules and bone substitute materials in bone regeneration.

A limitation of this study is that *in vitro* models do not fully reproduce the complexity of the *in vivo* biological environment. In addition, only a single melatonin concentration was evaluated, precluding the assessment of potential dose-dependent effects. The present study focused on early osteoblastic responses and did not evaluate later stages of osteogenic differentiation or extracellular matrix mineralization, which may respond differently to melatonin treatment. Furthermore, the use of a single human osteoblast-like cell line (SaOS-2) may limit the generalizability of the findings to other osteoblastic or primary cell models. Finally, the lack of direct assessment of osteoblastic differentiation markers and of potential biomaterial-melatonin interactions precludes mechanistic conclusions regarding the biological mechanisms underlying the observed responses.

Overall, under the present experimental conditions, melatonin alone did not differ significantly from the control group, whereas Bio-Oss®, either alone or combined with melatonin, was associated with reduced cell proliferation after 7 days. Cell viability was lower in the melatonin plus Bio-Oss® group at later time points, whereas type I collagen secretion remained comparable among all groups. The combination of melatonin with Bio-Oss® did not enhance early osteoblastic activity in this *in vitro* model. Additional investigations evaluating different melatonin concentrations, longer observation periods, and more complex experimental models are warranted to further elucidate the biological responses observed in the present study.

## 5 Conclusion

Within the limitations of this *in vitro* study, melatonin alone produced biological responses comparable to the control group, whereas Bio-Oss®, alone or combined with melatonin, was associated with reduced cell proliferation after 7 days. Cell viability was lower in the melatonin plus Bio-Oss® group at the final time point, while type I collagen secretion did not differ significantly among groups. These findings indicate that combining melatonin with Bio-Oss® did not enhance early osteoblastic activity under the experimental conditions evaluated in this study. Further studies are warranted to investigate the underlying mechanisms and to determine whether these findings can be reproduced under different experimental conditions.

## Ethical approval

This study was conducted using an established cell line and did not involve human participants or animals. Therefore, ethical approval was waived by the Research Ethics Committee of Faculdade São Leopoldo Mandic (protocol no. 2019/0184).

## Data availability statement

The data that support the findings of this study are available from the corresponding author upon reasonable request.

## Ethical statement

This study was conducted using an established cell line and did not involve human participants or animals. Therefore, ethical approval was not required. The use of the cell line was approved by the Research Ethics Committee of Faculdade São Leopoldo Mandic (protocol no. 2019/0184).

## Declaration of generative AI and AI-assisted technologies in the writing process

During manuscript preparation, the authors used ChatGPT and Grammarly for language editing. The authors, who take full responsibility for the final version, reviewed all content.

## Funding

This research did not receive any specific grant from funding agencies in the public, commercial, or not-for-profit sectors.

## Declaration of competing interest

The authors declare that they have no known competing financial interests or personal relationships that could have appeared to influence the work reported in this paper.
